# *bamSliceR*: cross-cohort variant and allelic bias analysis for rare variants and rare diseases

**DOI:** 10.1101/2023.09.15.558026

**Published:** 2023-09-17

**Authors:** Yizhou Peter Huang, Lauren Harmon, Eve Gardner, Xiaotu Ma, Josiah Harsh, Zhaoyu Xue, Hong Wen, Marcel Ramos, Sean Davis, Timothy J. Triche

**Affiliations:** a Michigan State University, East Lansing, MI, US; b Van Andel Institute, Grand Rapids, MI, US; c St. Jude Children’s Research Hospital, Memphis, TN, US; d Roswell Park Cancer Institute: Buffalo, NY, US; e University of Colorado Anschutz Medical Campus: Aurora, CO, US

## Abstract

Rare diseases and conditions create unique challenges for genetic epidemiologists precisely because cases and samples are scarce. In recent years, whole-genome and whole-transcriptome sequencing (WGS/WTS) have eased the study of rare genetic variants. Paired WGS and WTS data are ideal, but logistical and financial constraints often preclude generating paired WGS and WTS data. Thus, many databases contain a patchwork of specimens with either WGS or WTS data, but only a minority of samples have both. The NCI Genomic Data Commons facilitates controlled access to genomic and transcriptomic data for thousands of subjects, many with unpaired sequencing results. Local reanalysis of expressed variants across whole transcriptomes requires significant data storage, compute, and expertise. We developed the ***bamSliceR*** package to facilitate swift transition from aligned sequence reads to expressed variant characterization. ***bamSliceR*** leverages the NCI Genomic Data Commons API to query genomic sub-regions of aligned sequence reads from specimens identified through the robust Bioconductor ecosystem. We demonstrate how population-scale targeted genomic analysis can be completed using orders of magnitude fewer resources in this fashion, with minimal compute burden. We demonstrate pilot results from ***bamSliceR*** for the TARGET pediatric AML and BEAT-AML projects, where identification of rare but recurrent somatic variants directly yields biologically testable hypotheses. ***bamSliceR*** and its documentation are freely available on GitHub at https://github.com/trichelab/bamSliceR.

## Introduction

RNA sequencing (RNA-seq) captures transcriptomes with allelic resolution, and thereby can implicate functional genomic variants in disease. The underlying technology is now sufficiently mature to be a staple in research and clinical settings. A recent surge in RNA-seq data from clinical trials has overtaken whole genome sequencing (WGS) results in many settings as a primary tool for interrogating genome function and directly assessing expressed genomic variants^1^. For example, multiple Children’s Oncology Group trials have collected RNA-seq data as a biological correlative, often outpacing genome sequencing in the same populations^2^. With continued accrual, the number of patients with RNA-seq data has nearly tripled, without a concomitant increase in WGS data^3^. Combining results from WGS and WTS can maximize variant yield for rare diseases.

In 2019, the Leucegene project recognized this opportunity, and developed a local assembly approach to identify mutations in RNA-seq data^4^. This method is computationally efficient relative to prior whole genome variant calling algorithms, but it involves a re-alignment process and does not take full advantage of the standardized Binary Alignment and Mapping (BAM) format^5^ supported by cloud-based repositories such as the NCI Genomic Data Commons (GDC). The GDC is an important resource of harmonized sequencing data from large translational research consortia, including The Cancer Genome Atlas^6^ (TCGA), TARGET^2^, and BEAT-AML^7^. The GDC Application Programming Interface (API) allows authorized users to query aligned data via genomic ranges or slices^8^. This provides an efficient basis for pipelines and tools facilitating rapid exploration, annotation, and experimental validation of genetic lesions in rare diseases.

Here, we present an R/Bioconductor software package (***bamSliceR***) for automatically and efficiently extracting coordinate- or range-based BAM reads from targeted genomic regions, and from large RNA-seq cohorts. bamSliceR bypasses the historically tedious variant calling workflow, thereby reducing time, space, and computational burden by orders of magnitude relative to standard methods. Users can quickly transition from raw alignment data to variant characterization, and perform population-scale targeted genomic inspections. We leverage RNA-seq data from more than 3,000 subjects to demonstrate how this lightweight workflow can expand sample size for rare variants and diseases, increasing scale and scope for discovery and validation.

## Methods

**Figure 1.**
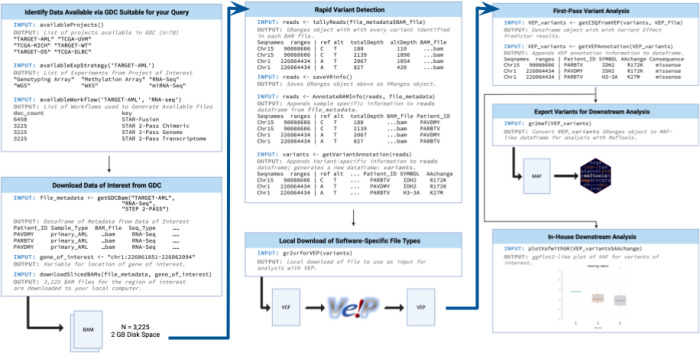
bamSliceR Workflow and Functionality for querying, downloading, and annotating BAM files.

The ***bamSliceR*** package has four basic functions, as illustrated in Figure 1. The first function integrates the GDC API within the ***bamSliceR*** R statistical programming environment, which enables users to query databases, remotely BAM-slice genomic regions of interest, and automatically import those sequences for local alignment. *bamSliceR* generates a metadata frame for each BAM file, which enables users to custom-filter the data based on sample type(s), sequencing type(s) (RNA-seq/WGS), or other criteria. This enhances the user’s ability to tailor the analysis to their specific needs. Next, ***bamSliceR*** uses gmapR^9^ and VariantTools^10^ to tally coverage and counts of variant alleles. ***bamSliceR*** then estimates the Variant Allele Fraction (VAF) for each mutation, and applies VariantAnnotation^11^ to predict associated amino acid changes. This allows parallel computing and accelerated analysis time for users with access to a High-Performance Computing (HPC) cluster or cloud. Annotation of predicted variant consequence is delegated to the Ensembl Variant Effect Predictor (VEP). Annotated variants can be exported to VCF, VRanges objects, or Mutation Annotation Format (MAF)^12^. ***bamSliceR*** also incorporates hooks for downstream analysis and visualization (co-occurrence, oncoplots, lollipop diagrams for protein hotspots, and survival analysis with Kaplan-meier plots).

## Results

### Oncohistone variants in pediatric leukemia

Pediatric acute myeloid leukemia (AML) is a genetically heterogeneous and often lethal disease^13^. Most cases reveal few if any actionable sequence variants, with a remarkably low mutation burden and a preponderance of diverse structural variants^14^. Nevertheless, molecular features can drive treatment decisions. For example, most patients with *DEK::NUP214* fusions harbor co-occurring *FLT3* internal tandem duplications. Targeting this aberration revealed the immunogenicity of the fusion, and when combined with stem cell transplantation, has improved 5-year survival from under 10% to over 85% in young patients^15^. Unfortunately, identifying actionable sequence variants from WGS is challenging both technically and statistically, as the population of AML patients is small relative to common diseases. Characterized pediatric cases with WGS number in the low hundreds. The analysis of expressed sequence variants from RNA-seq reads is therefore quite attractive.

Mutations of histone H3.3 lysine 27 (H3K27) to methionine (M, H3K27M) were originally documented in high-grade midline glioma,^16^ where they frequently accompany *TP53* mutations. More recently, mutations of H3.1 K27 to isoleucine (I) or methionine (H3K27I/M) were documented in adult AML patients^17^ and in pre-leukemic stem cells from patients who went on to develop secondary AML^18^. However, the genetic context, age groups (histone mutations have not previously been reported in pediatric AML patients), mechanism, and clinical impact of H3K27 mutations in myeloid leukemogenesis remains poorly understood at best.

**Figure 2.**
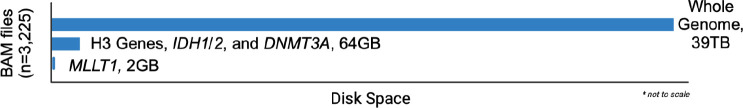
Reduction of Disk Space Required for Analysis by BamSlicing. The 3,225 RNAseq BAM files obtained from 2,281 subjects in TARGET-AML are approximately 39TB. Using bamSliceR to slicing the reads covering exons of 11 Histone 3 genes (*H3F3A, HIST1H3A, HIST1H3H, HIST1H3I, HIST1H3J, HIST1H3B, HIST1H3C, HIST1H3D, HIST1H3E, HIST1H3F, HIST1H3G*) and genes encoding epigenetic factors (*IDH1/2, DNMT3A, RUNX1, ASXL1/2, TET1/2*) that collectively span 152,026 bp of non-contiguous genomic space reduced the total size of BAM files to 62GB. Furthermore, slicing the reads covering a single Human *ENL* gene span of 73,594 bp only requires 2GB of total disk space for 3,225 BAM files.

To investigate the genetic landscape of H3K27M in AML across age groups, we examined 11 histone 3 genes as well as genes encoding epigenetic factors that are frequently mutated in AML patients (*IDH1/2*, *DNMT3A*, *RUNX1*, *ASXL1/2*, and *TET1/2*). These genes collectively span 152,026 bp of non-contiguous genomic space. Using the *bamSliceR* pipeline, we automatically processed 3,225 and 735 RNA-seq BAM files obtained from 2,281 and 653 subjects in the TARGET-AML (pediatric) and BEAT-AML (adult) cohorts, respectively. This step alone reduced the total size of the BAM files of the TARGET-AML cohort from 39TB to 62GB (Fig. 2), while still retaining all the essential genetic information required to perform an epidemiological study of these rare AML mutations. We identified 9 pAML and 7 adult AML patients that harbored a K27M mutation, with multiple lines of evidence based on Variant Allele Frequency (VAF >0.15), total read depth (>8), and WGS data where available (Supplementary Table S1). We found H3K27M mutations on both replication-coupled H3.1 and replication-independent H3.3, with most mutations occurring in the H3.1 gene (Supplementary Table S1). The incidence of H3K27M in pediatric AML is lower (~0.3 %) than in adult AML (~0.8%; Supplementary Table S2), and we confirmed the existence of H3K27 variants in normal karyotype induction failure patients (Figure 4).

**Figure 3.**
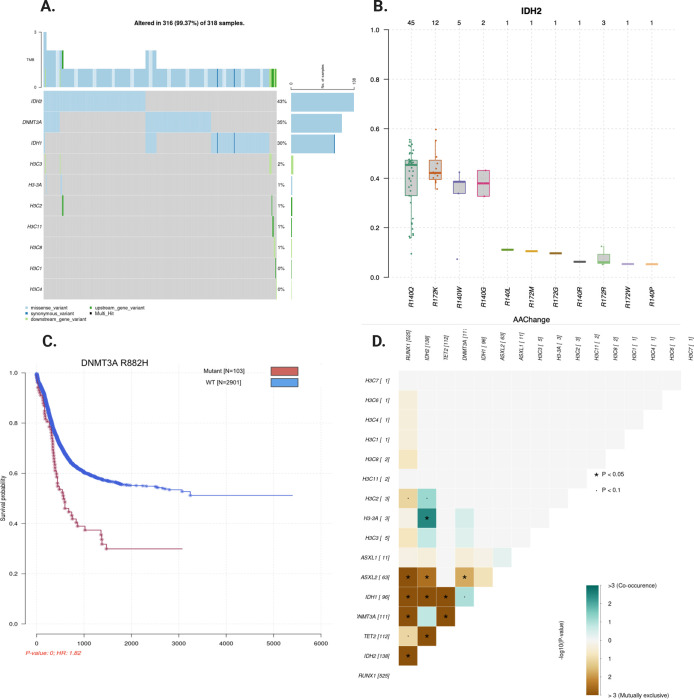
*BamSIlcing* Data Visualization Functionality. **A-D** Example of automatically generation of *Oncoplot*, *survival analysis, VAF distribution* and *Mutual Exclusivity analysis.* **B**. VAF plotting of different mutations of *IDH2* at either *R140* or *R172* ordering by median of VAF. *R140Q* and *R172K* are most prevalent and *R172K* mutation are always clonal which are usually have mean allele frequency around ~50% assuming pure sample (VAF plotting of *H3K27M* and *DNMT3A* are in Supplementary Figure S1 and S2). **C**. Kaplan meier curve by grouping samples based on mutation status (WT vs. DNMT3A) in both TARGET and BEAT AML cohorts (n = 2934). **D.** Mutual exclusivity plot by pair-wise Fisher’s Exact test detected *K27M* and *IDH2 R172K* are co-occuring mutations (p < 0.0032).

**Figure 4.**
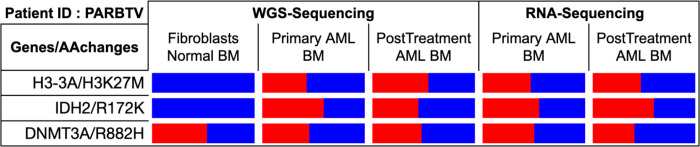
Clonal mutations observed in TARGET AML subject PARBTV from WGS and RNAseq Data. Variant allele frequency of mutations *H3F3A K27M, IDH2 R172K* (somatic), and *DNMT3A R882H* (germline) of a pediatric patient from the TARGET-AML cohort with both RNAseq and WGS data from two timepoints (diagnosis and after treatment) (BLUE: allele frequency of WT; RED: allele frequency of mutant).

We used ***bamSliceR*** to generate VAF distribution plots for each mutation and estimate their clonal status. For example, we see that *IDH2* ^R172K^ and *DNMT3A*^R132H^ mutations are persistent clonal events (VAF ~50%) in the pAML cohort, that were previously believed to only occur in adult AML patients (Fig 3C; Supplementary Fig S1). We discerned that the K27M mutation in *H3C2*, *H3C3*, *H3C4*, *H3C11* (H3.1) and *H3F3A* (H3.3) genes are always clonal (Supplementary Fig S2), consistent with their occurrence in pediatric high-grade glioma. Oncoplots and mutual exclusivity analysis showed that *H3F3A* K27M (H3.3) and *IDH2* mutations always co-occur (p<0.05). Interestingly, two pAML patients harboring *H3F3A* K27M and *IDH2*^R172K^ mutations failed induction therapy, suggesting that the two mutations may synergize to cause chemoresistance. For pAML patients where RNA-seq and WGS data are both available, we used bamSliceR to confirm that the somatic *H3F3A* K27M and *IDH2*^R172K^ mutations are constantly expressed at high levels, with low allelic bias, consistent with DNA sequencing results (Supplementary Table S1; Fig 4F). To facilitate in-depth studies of patients with samples at multiple disease stages, ***bamSliceR*** includes functionality to identify and subset the matched subjects and data files. Using this function, we found that one pAML patient with somatic *H3F3A* K27M and *IDH2*^R172K^ mutations also harbored a germline *DNMT3A*^R882C^ mutation throughout disease progression (Fig 4).

Taken together, these data suggest that H3K27M is a clonal mutation that may synergize with metabolic and epigenetic variants (e.g. *IDH2*^R172K^ and *DNMT3A*^R882C^) to drive aggressive and refractory pAML. *IDH* mutations have been thought mutually exclusive with *H3K27* variants. Not only do they co-occur, *IDH2* mutations are in fact enriched for *H3K27* mutant cases. By using RNA-seq data to expand our sample size and automating the alignment and variant calling process in ***bamSliceR***, we document statistically and clinically significant co-occurrence of oncometabolic *IDH2* variants with high-risk *H3K27* pAML mutant cases, yielding testable hypotheses and new translational avenues^19^ for a subset of AML patients at high risk of treatment failure.

### *MLLT1* YEATS domain indels in pediatric tumors

The ENL protein (encoded by the *MLLT1* gene) is a subunit of the super elongation complex (SEC) involved in transcriptional elongation during early development. Small in-frame insertion-deletion (indel) mutations within the YEATS domain of *MLLT1* were first identified in Wilms tumors^20^. Further work revealed that indels in the YEATS domain alter chromatin states, dysregulating cell-fate control and driving tumorigenesis^21^. These same indels can transform hematopoietic cells by mitigating polycomb silencing, but only one case of pediatric AML that harbored this type of mutation had previously been documented (http://cancer.sanger.ac.uk/cosmic).

**Figure 5.**
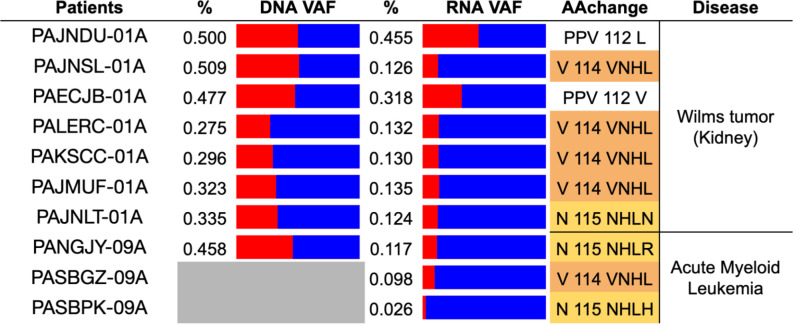
Analysis of Rare *ENL* YEATS Domain Mutations in pAML patients. Allelic bias based on Variant Allele Frequency of in-frame insertion mutations in Human *ENL* gene of Wilms Tumor and AML patients with both RNAseq and WGS data.

As a second case study for the efficacy of our package, we used ***bamSliceR*** to analyze the TARGET RNA-seq and WGS data for any evidence of ENL (*MLLT1*) YEATS domain mutations. Using our pipeline, we discovered three pAML patients carrying mutations in *ENL*-YEATS, with one pAML patient exhibiting allelic bias similar to that observed in Wilms tumor patients (Fig 5). We also identified putative indel mutations near the YEATS domain that have not been detected in Wilms tumor patients (Supplementary Table S3). These new results lead to the biologically testable hypothesis that *ENL*-YEATS mutations primarily upregulate *HOXA* gene expression in pAML, similar to their role and function in favorable histology Wilms tumors^22^

## Discussion

We developed ***bamSliceR*** to address two practical challenges: resource-sparing identification of candidate subjects, and variant detection from aligned sequence reads, across thousands of controlled-access subjects. The GDC BAM Slicing API (https://docs.gdc.cancer.gov/API/Users_Guide/BAM_Slicing/) is a practical and well documented REST API for this purpose. Yet, perhaps due to the challenge of the former task, we find relatively little published work referencing usage of the GDC API, even in studies where specific candidate variants are evaluated. Instead, reliance upon variant calls from existing studies, or transfer and recall of variants from raw sequence data, is often documented. The former assumes a single best method for variant detection fits all experimental designs (an assumption contradicted by many benchmarks[*cite*]). The latter is grossly inefficient.

In clinical genetic analysis, direct evaluation of fragment-level evidence for a candidate variant is routine, regardless of the confidence level a variant calling pipeline may assign to a putative genetic variant. The same raw material is available via controlled access across many population-scale projects representing billions of dollars in public funding. The relatively simple toolkit we provide here extends this practice in an efficient and user-friendly way to the Bioconductor ecosystem, significantly expanding the pool of potential subjects for rare variant detection in rare diseases. Importantly, the same well-documented API implemented by the GDC is feasible for Common Fund datasets, such as the Gabriella Miller Kids First! (GMKF) and INCLUDE projects, and can be further extended to transcriptome-indexed reads as well as open access resources such as SRA.

Support for efficient retrieval of range-based queries is a key feature of the SAM/BAM format and htslib/htsget implementations. Authentication and authorization create challenges for straightforward usage in controlled access data, but as the GDC API and its downstream users illustrate, this challenge can be overcome, and is increasingly important as projects with whole-genome sequence data from population-scale biobanks emerge. We present bamSliceR as a concrete example of what can be accomplished by wedding clinical and genomic data management processes to an efficient, standardized API. Our hope is that it will enable users to perform previously challenging evaluation of raw data evidence for genetic and genomic variants at scale, and that user uptake will spur further expansion of support for coordinate-based queries of sequence databases.

## Supplementary Material

Supplement 1

## References

[1] Casamassimi A., Federico A., Rienzo M., Esposito S. & Ciccodicola A. Transcriptome Profiling in Human Diseases: New Advances and Perspectives. Int. J. Mol. Sci. 18, (2017). 10.3390/ijms18081652 PMC5578042 28758927

[2] Bolouri H. The molecular landscape of pediatric acute myeloid leukemia reveals recurrent structural alterations and age-specific mutational interactions. Nat. Med. 24, 103–112 (2018). 29227476 10.1038/nm.4439 PMC5907936

[3] Farrar J. E. Long Noncoding RNA Expression Independently Predicts Outcome in Pediatric Acute Myeloid Leukemia. J. Clin. Oncol. JCO2201114 (2023). 10.1200/JCO.22.01114 PMC10414715 36795987

[4] Audemard E. O. Targeted variant detection using unaligned RNA-Seq reads. Life Sci Alliance 2, (2019). 10.26508/lsa.201900336 PMC6701478 31427380

[5] Li H. The Sequence Alignment/Map format and SAMtools. Bioinformatics 25, 2078–2079 (2009). 19505943 10.1093/bioinformatics/btp352 PMC2723002

[6] Cancer Genome Atlas Research Network. Genomic and epigenomic landscapes of adult de novo acute myeloid leukemia. N. Engl. J. Med. 368, 2059–2074 (2013). 23634996 10.1056/NEJMoa1301689 PMC3767041

[7] Tyner J. W. Functional genomic landscape of acute myeloid leukaemia. Nature 562, 526–531 (2018). 30333627 10.1038/s41586-018-0623-z PMC6280667

[8] Wilson S. Developing Cancer Informatics Applications and Tools Using the NCI Genomic Data Commons API. Cancer Res. 77, e15–e18 (2017). 29092930 10.1158/0008-5472.CAN-17-0598 PMC5683428

[9] Wu T. D. & Nacu S. Fast and SNP-tolerant detection of complex variants and splicing in short reads. Bioinformatics 26, 873–881 (2010). 20147302 10.1093/bioinformatics/btq057 PMC2844994

[10] Lawrence M. & Gentleman R. VariantTools: an extensible framework for developing and testing variant callers. Bioinformatics 33, 3311–3313 (2017). 29028267 10.1093/bioinformatics/btx450 PMC5860039

[11] Obenchain V. VariantAnnotation: a Bioconductor package for exploration and annotation of genetic variants. Bioinformatics 30, 2076–2078 (2014). 24681907 10.1093/bioinformatics/btu168 PMC4080743

[12] Mayakonda A., Lin D.-C., Assenov Y., Plass C. & Koeffler H. P. Maftools: efficient and comprehensive analysis of somatic variants in cancer. Genome Res. 28, 1747–1756 (2018). 30341162 10.1101/gr.239244.118 PMC6211645

[13] Gruber T. A. An Inv(16)(p13.3q24.3)-encoded CBFA2T3-GLIS2 fusion protein defines an aggressive subtype of pediatric acute megakaryoblastic leukemia. Cancer Cell 22, 683–697 (2012). 23153540 10.1016/j.ccr.2012.10.007 PMC3547667

[14] Bolouri H. A B-cell developmental gene regulatory network is activated in infant AML. PLoS One 16, e0259197 (2021). 34793513 10.1371/journal.pone.0259197 PMC8601427

[15] Tarlock K. Significant Improvements in Survival for Patients with t(6;9)(p23;q34)/DEK-NUP214 in Contemporary Trials with Intensification of Therapy: A Report from the Children’s Oncology Group. Blood 138, 519 (2021).

[16] Schwartzentruber J. Driver mutations in histone H3.3 and chromatin remodelling genes in paediatric glioblastoma. Nature 482, 226–231 (2012). 22286061 10.1038/nature10833

[17] Lehnertz B. H3 K27M/I mutations promote context-dependent transformation in acute myeloid leukemia with RUNX1 alterations. Blood 130, 2204–2214 (2017). 28855157 10.1182/blood-2017-03-774653

[18] Boileau M. Mutant H3 histones drive human pre-leukemic hematopoietic stem cell expansion and promote leukemic aggressiveness. Nat. Commun. 10, 1–12 (2019). 31253791 10.1038/s41467-019-10705-z PMC6599207

[19] Thomas D. Dysregulated Lipid Synthesis by Oncogenic IDH1 Mutation Is a Targetable Synthetic Lethal Vulnerability. Cancer Discov. 13, 496–515 (2023). 36355448 10.1158/2159-8290.CD-21-0218 PMC9900324

[20] Perlman E. J. MLLT1 YEATS domain mutations in clinically distinctive Favourable Histology Wilms tumours. Nat. Commun. 6, 1–10 (2015). 10.1038/ncomms10013 PMC4686660 26635203

[21] Wan L. Impaired cell fate through gain-of-function mutations in a chromatin reader. Nature 577, 121–126 (2020). 31853060 10.1038/s41586-019-1842-7 PMC7061414

[22] Gadd S. A Children’s Oncology Group and TARGET initiative exploring the genetic landscape of Wilms tumor. Nat. Genet. 49, 1487–1494 (2017). 28825729 10.1038/ng.3940 PMC5712232

