# Supplementary material for: bamSliceR: cross-cohort variant and allelic bias analysis for rare variants and rare diseases
Source: bioRxiv. 2023 Sep 17:2023.09.15.558026. Preprint. [Version 1] doi: 10.1101/2023.09.15.558026 (PMC10516001; doi:10.1101/2023.09.15.558026)

bioRxiv preprint doi: https://doi.org/10.1101/2023.09.15.558026; this version posted September 17, 2023. The copyright holder for this preprint (which was not certified by peer review) is the author/funder, who has granted bioRxiv a license to display the preprint in perpetuity. It is made available under aCC-BY 4.0 International license.

Supplemental data

(Each table should be a separate excel file.)

Table S1

Co-occurence status of *H3K27M* and *IDH2* mutations in TARGET-AML and BEAT-AML.
Identification of 9 pAML patients that harboared K27M mutation on Histone 3 genes based on evidence of VAF > 0.15 and total read depth > 8 and whether the mutation can be captured by both RNA-seq and WGS data. Co-occurence of *H3F3A* (H3.3) *K27M* and *IDH2 R172K* are shown in 2 pAML patients. Co-occurence of *H3K27M* (H3.3 & H3.1) and *IDH2 R140Q/R172K* are shown in 5 adult AML patients.

		H3K27M					IDH2				
	**CASE_ID**	**SYMBOL**	**DNA-vaf-T1**	**DNA-vaf-T2**	**RNA-vaf-T1**	**RNA-vaf-T2**	**SYMBOL**	**DNA-vaf-T1**	**DNA-vaf-T2**	**RNA-vaf-T1**	**RNA-vaf-T2**
**H3.3**	PARBTV	H3F3A	47%	48%	47%%	47%	R172K	38%	48%	56%	59%%
	PAVDMY	H3F3A			49%	47%/47%	R172K			39%	40%/48%
**H3.1**	PAPVGE	HIST1H3C			58%%		-			-	
	PAUZTH	HIST1H3J(K27I)			0%	38%%	-			-	-
	PAUUPR	HIST1H3I			0%	35%%	-			-	-
	PAKWCU	HIST1H3D			17%%		-			-	
	PAXFAG	HIST1H3C			54%%		-			-	
	PAXKAL	HIST1H3I			50%		-			-	
	PATFGK	HIST1H3C			50%		-			-	

		H3K27M				IDH2			
	**CASE_ID**	**SYMBOL**	**DNA-vaf**	**RNA-vaf**	**TS-vaf**	**SYMBOL**	**DNA-vaf**	**RNA-vaf**	**TS-vaf**
**H3.3**	2148	H3F3A	29%	47%		R140Q	40%	52%	
**H3.1**	2354	HIST1H3C	34%/33%	low-exp		R172K	36%/31%	47%	
	2429	HIST1H3B	33%/47%	35.29%/100%		-	-	-	
	2498	HIST1H3C	37%	38%		R140Q	48%	47%	
	2530	HIST1H3	40%	low-exp		R172K	46%	49%	

bioRxiv preprint doi: https://doi.org/10.1101/2023.09.15.558026; this version posted September 17, 2023. The copyright holder for this preprint (which was not certified by peer review) is the author/funder, who has granted bioRxiv a license to display the preprint in perpetuity. It is made available under aCC-BY 4.0 International license.

		B							
	2611	HIST1H3 D		58%	39%	-		-	-
	2721	HIST1H3 B			36%	R172K			36%

bioRxiv preprint doi: https://doi.org/10.1101/2023.09.15.558026; this version posted September 17, 2023. The copyright holder for this preprint (which was not certified by peer review) is the author/funder, who has granted bioRxiv a license to display the preprint in perpetuity. It is made available under aCC-BY 4.0 International license.

Table S2

***H3K27* variants: published work (n=1049) and TARGET/BEAT-AML cohorts (n = 2934, via bamSliceR).**
Lehnertz et al. *Blood 2017* documented 2 adult AML patients. Boileau et al. *Nat Commun 2019* documented 4 adult AML patients. We documented 16 AML patients from TARGET and BEAT AML cohorts.

Cohort	Cohort Size (n)	WGS/WXS (n)	RNAseq (n)	DNA Methylation (n)	H3K27M/I (%/n)
**Lehnertz et al. *Blood* (2017)**					
**Leucegene**	415		415		0.48%/2
**Boileau et al. *Nat Commun* (2019)**					
**Toronto**	312	312			0.64%/2
**Lebanon**	122	122			0.8%/1
**TCGA**	200	200	200	200	0.5%/1
**Identification of H3K27M in BEAT-AML and TARGET-AML**					
**TARGET 20/21**	2045	365	**2281**	2000	0.4%/9
**Beat-AML**	826	798	**653**		0.8%/7

bioRxiv preprint doi: https://doi.org/10.1101/2023.09.15.558026; this version posted September 17, 2023. The copyright holder for this preprint (which was not certified by peer review) is the author/funder, who has granted bioRxiv a license to display the preprint in perpetuity. It is made available under aCC-BY 4.0 International license.

Table S3

Human *MLLT1* YEATS domain insertion/deletion variants identified in the pan-TARGET cohort.

Chromosome	POS	SYMBOL	AAchange	REFCODON	VARCODON	REFAA	VARAA	alt_count	totla_count	VAF	patient_id
**chr19**	6230649	MLLT1	V114VNHL	GTG	GTGAACCACCTG	V	VNHL	5	51	0.09804	PASBGZ
**chr19**	6230642	MLLT1	H116HLRP	CAC	CACCTGCGCCCC	H	HLRP	2	460	0.00435	PANGJY
**chr19**	6230645	MLLT1	N115NPLR	AAC	AACCCCCTGCGC	N	NPLR	4	470	0.00851	PANGJY
**chr19**	6230645	MLLT1	N115NHLR	AAC	AACCACCTGCGC	N	NHLR	55	470	0.11702	PANGJY
**chr19**	6230640	MLLT1	HL116L	CACCTG	CTG	HL	L	2	134	0.01493	PALHVV
**chr19**	6230646	MLLT1	N115NHLH	AAC	AACCACCTGCAC	N	NHLH	2	76	0.02632	PASBPK
**chr19**	6230582	MLLT1	LL135L	CTCCTG	CTG	LL	L	2	175	0.01143	PAUMUZ
**chr19**	6230611	MLLT1	NP126P	AACCCC	CCC	NP	P	2	370	0.00541	PAUHGM
**chr19**	6230616	MLLT1	TFN123N	ACCTTCAAC	AAC	TFN	N	2	48	0.04167	PAWVPZ
**chr19**	6230620	MLLT1	TF123F	ACCTTC	TTC	TF	F	2	690	0.00290	PAVDXR
**chr19**	6230574	MLLT1	AG138G	GCCGGC	GGC	AG	G	2	175	0.01143	PABYYR
**chr19**	6230570	MLLT1	GG139G	GGCGGG	GGG	GG	G	2	1047	0.00191	PAUWZR

bioRxiv preprint doi: https://doi.org/10.1101/2023.09.15.558026; this version posted September 17, 2023. The copyright holder for this preprint (which was not certified by peer review) is the author/funder, who has granted bioRxiv a license to display the preprint in perpetuity. It is made available under aCC-BY 4.0 International license.

Figure S1

VAF distribution of DNMT3A variants.

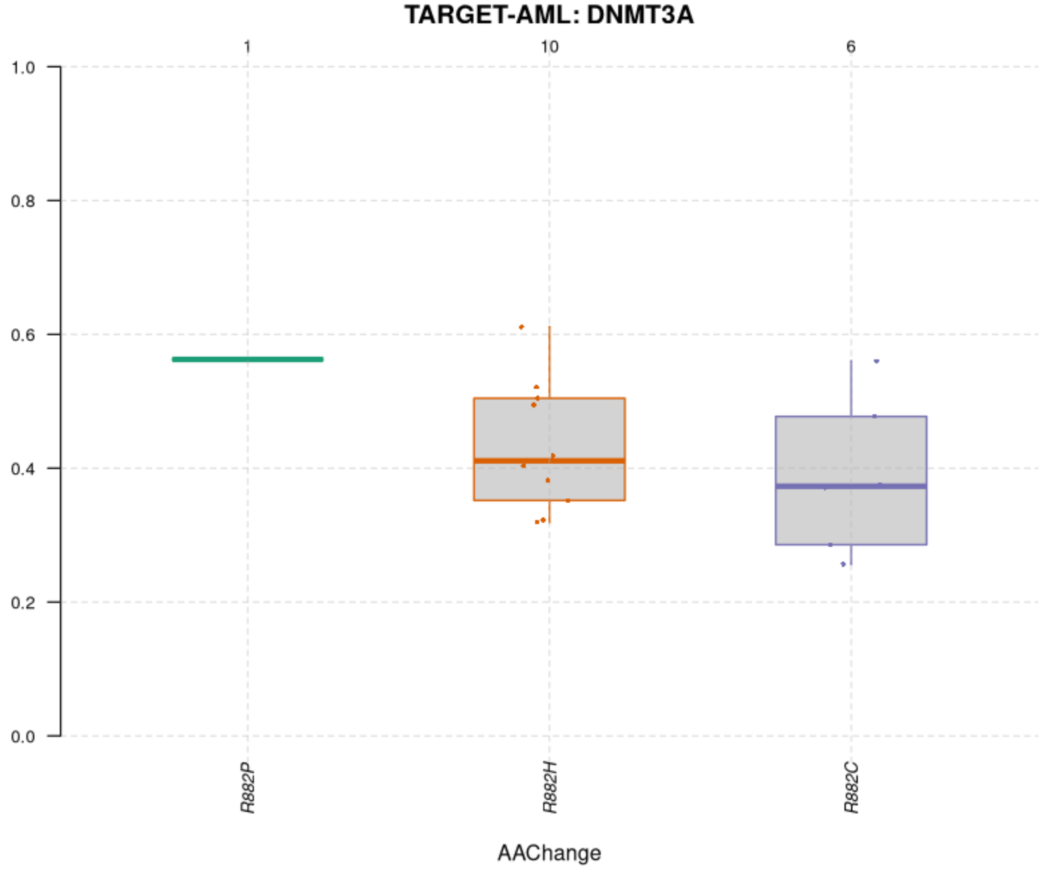

bioRxiv preprint doi: https://doi.org/10.1101/2023.09.15.558026; this version posted September 17, 2023. The copyright holder for this preprint (which was not certified by peer review) is the author/funder, who has granted bioRxiv a license to display the preprint in perpetuity. It is made available under aCC-BY 4.0 International license.

Figure S2

VAF distribution of H3K27 variants.

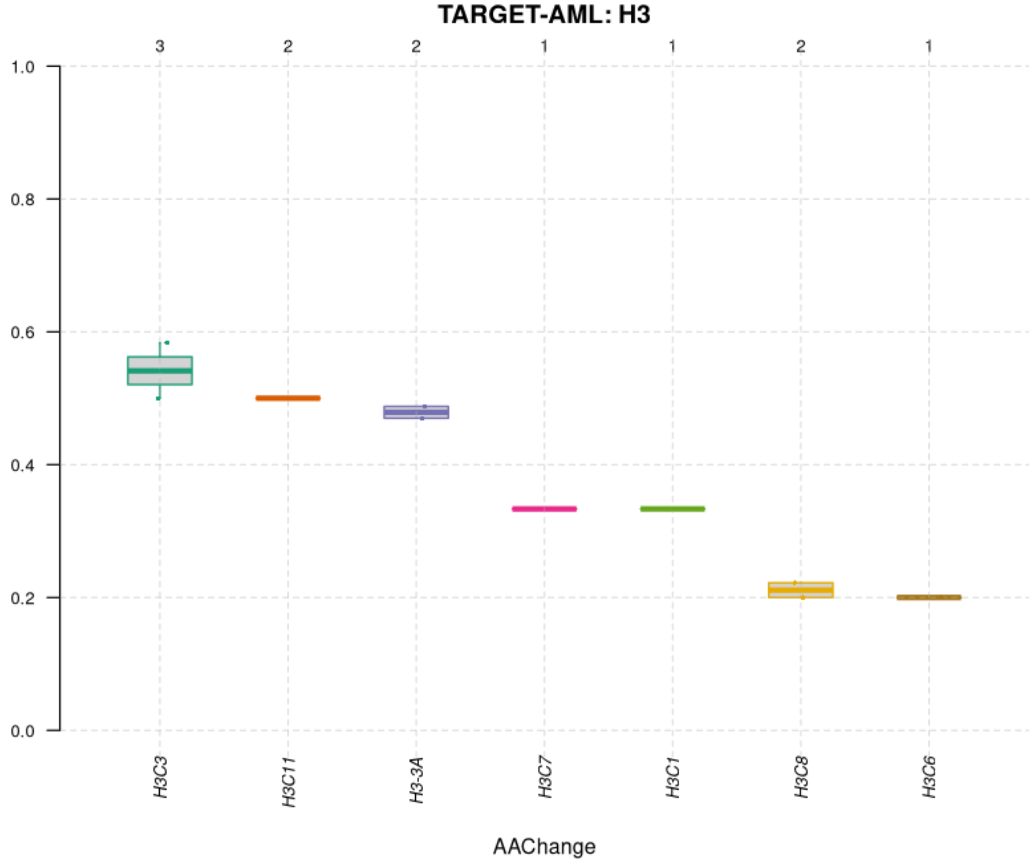

Supplement: Supplementary file 1 [file NIHPP2023.09.15.558026v1-supplement-1.pdf]
